# Acoustic Velocity Measurement for Enhancing Laser UltraSound Imaging Based on Time Domain Synthetic Aperture Focusing Technique

**DOI:** 10.3390/s23052635

**Published:** 2023-02-27

**Authors:** Taeil Yoon, Younggue Kim, Muhammad Awais, Byeongha Lee

**Affiliations:** School of Electrical Engineering and Computer Science, Gwangju Institute of Science and Technology, 123 Cheomdangwagi-ro, Buk-gu, Gwangju 61005, Republic of Korea

**Keywords:** laser ultrasound, time domain synthetic aperture focusing technique, acoustic velocity measurement, 3 × 3 optical interferometer

## Abstract

A method to enhance laser ultrasound (LUS) image reconstruction with the time-domain synthetic aperture focusing technique (T-SAFT) is presented, in which the acoustic velocity is extracted in situ with curve fitting. The operational principle is provided with the help of a numerical simulation, and the confirmation is provided experimentally. In these experiments, an all-optic LUS system was developed by using lasers for both excitation and detection of ultrasound. The acoustic velocity of a specimen was extracted in situ by fitting a hyperbolic curve to its B-scan image. The needle-like objects embedded within a polydimethylsiloxane (PDMS) block and a chicken breast have been successfully reconstructed using the extracted in situ acoustic velocity. Experimental results suggest that knowing the acoustic velocity in the T-SAFT process is important not only in finding the depth location of the target object but also for producing a high resolution image. This study is expected to pave the wave to the development and usage of all-optic LUS for bio-medical imaging.

## 1. Introduction

The laser ultrasound (LUS) technique has been widely applied to non-destructive testing for structural health monitoring and tomographic imaging [1,2,3,4,5]. The LUS uses a laser to generate the ultrasound for imaging. This is unlike conventional ultrasound techniques that use ultrasonic transducers. By directing a laser pulse to the surface of a specimen, a short packet of ultrasound wave is generated without physically contacting the specimen. The generated ultrasound propagates into the volume of the specimen and is reflected or scattered by the target objects within the volume. The returning ultrasound, called the echo ultrasound, is generally monitored by an ultrasonic transducer at the surface of the specimen. Since the echo signal returning from a deeper object arrives later, we can get the distribution of the target objects along the depth by measuring the echo signals in terms of time (the A-scan image). By performing the same A-scan measurements while scanning the measuring point laterally and placing the A-scan images side by side, we can obtain a 2-dimentional distribution of the target object (the B-scan image). However, measuring the echo ultrasound with a conventional transducer loses most of the benefits of the LUS technique, especially the non-contact modality. The conventional method requires physical contact with the specimen or impedance matching material [1,2].

In a recent study, the tissue layer structure of a human arm was LUS imaged at a depth of several centimeters [6]. The generation of the ultrasound wave was conducted with a laser and the echo ultrasound was measured with a laser Doppler velocity meter (LDV). Since the excitation and the detection of ultrasound were produced with lasers, this procedure can be called all-optic LUS. However, the LDV system is bulky and high cost, in general. It is thought that the optical measurement of ultrasound can be conducted by using various other optical interferometers. One of the most feasible and effective candidates is the multi-port interferometer, that has more than three interference arms. With the inherent phase shift occurring at each port on the multi-port interferometer, the acoustically-induced surface displacement or vibration can be measured in a nanometer resolution with its direction of moving [7,8].

In the case of an all-optic LUS system, however, the imaging resolution is limited due to the divergence of the acoustic wave. The ultrasound wave excited at a point on the specimen surface expands spherically when it propagates within the volume of the specimen. The echo ultrasound reflected or scattered by a target object in the specimen expands back to the surface. Since both the excitation and the detection are conducted only at the surface in LUS, the acoustic wave divergence is inevitable unless an acoustic lens is used. The effect of an acoustic lens can be obtained without the use of additional hardware by image reconstruction software. The time-domain synthetic aperture focusing technique (T-SAFT) [9,10,11,12,13,14,15,16,17,18,19,20] measures the echo ultrasound returning from a target object at various positions on the specimen surface. By coherently adding the echo signals to proper phases, only the signal returning from a particular target point can be selected or synthetized, similar to the function of acoustic wave focusing. By controlling the phases, the focal length of the synthetic lens can be adjusted. In other words, we can select or collect the acoustic waves returning from just the specific position within the volume of specimen. However, knowing the acoustic velocity within the specimen is essential to properly utilize the synthetic aperture technique. When the velocity is not accurate enough, there is considerable distortion in the image reconstructed by using T-SAFT [9,10,11].

In ultrasound (US) medical imaging, Anderson et al. argued that the actual acoustic velocity within a specimen was an important factor, and a method of estimating the acoustic velocity through curve fitting was introduced [9,10]. However, it was a study performed by using a conventional contact-type transducer and it did not show the image reconstruction by using T-SAFT. Also, the findings had not yet been applied to the field of all-optic LUS imaging. Jaeger et al. calculated the acoustic velocity in the medium by measuring the angle-dependent aberration delay [21,22,23]. Napolitano et al. studied the method of finding the optimal acoustic velocity by analyzing the lateral spatial frequency of the images reconstructed with several trial sound speeds [24]. However, these methods were time consuming and not convenient because they measured at multiple angles or selected the optimal value from the reconstruction results obtained using various acoustic velocities. Recently, methods for measuring acoustic velocity based on deep learning models have been introduced for photoacoustic (PA) imaging [25]. However, this requires images for each acoustic velocity in order to construct a training dataset, and the implementation is accompanied by complicated processes.

In this study, we propose an improved all-optic LUS system that can more accurately reconstruct the distribution of target objects within the volume of a specimen. Both the excitation and the detection of ultrasound waves are conducted with lasers. The acoustic velocity is extracted in situ with the B-scan image of a needle-like object by hyperbola curve fitting. Then, the initial distribution of target objects is reconstructed using the T-SAFT algorithm equipped with the extracted velocity. The proof of concept is presented with numerical simulations and the experimental confirmation is conducted using the LUS imaging of the objects embedded in a polydimethylsiloxane (PDMS) block and a chicken breast. To the best of our knowledge, the acoustic velocity measurement based on the T-SAFT curve fitting and its usage for enhancing the LUS image reconstruction have not been proposed yet in the field of all-optic LUS imaging.

## 2. Materials and Methods

### 2.1. Time-Domain Synthetic Aperture Focusing Technique (T-SAFT)

In an ideal LUS system, a short packet of ultrasound wave is generated at a point on the surface of a specimen and its echo waves are detected at the same point, forming an A-scan image. By performing a line-scan along the surface of the specimen, a B-scan image is constructed. If the acoustic wave propagates along the depth direction like a narrow ray, the B-scan image will show the distribution of the target objects directly and without confusion. However, both the excitation and the echo ultrasound waves are spherically diverging, meaning that the signal from a single target point can be detected at many different positions across the surface. Therefore, it is not easy to determine the location of the target object directly without using an acoustic lens. In other words, the B-scan image of a point target is not a point anymore but distributed in general.

However, with the phases of the echo signals measured at various surface positions, we can obtain the 3-dimensional location of the point within the volume. In this case, the phase is related to the time delay of the echo signal of the point in each A-scan image. The more widely the echo signals are distributed, the more precisely the location of the target point can be specified. On the other hand, since the echo signals from a point target form a curve in a B-scan image, we can collect the echo signals from the target point by adding the signals along that curve. The adding along a curve, or a curved area for a 2-dimensional case, is the same as the function of a focusing lens; as such, it is called the synthetic aperture focusing technique (SAFT). Further, as the phase is counted using the time delay, the process is called the time-domain SAFT or T-SAFT. The SAFT can be produced in the spatial frequency domain [17,20,26,27], however this is beyond the scope of this work.

**Figure 1:**
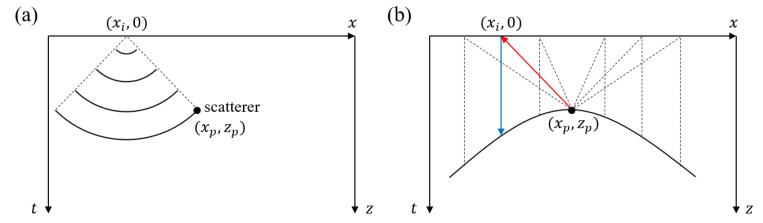
Formation of a hyperbola shape curve in a B-scan image. (**a**) The acoustic wave generated at the surface $\left(x_{i},\;0\right)$ propagates into the specimen with a spherical wavefront and then spherically returns from $\left(x_{p},\;z_{p}\right)$. (**b**) The time delays plotted with respect to the lateral scanning positions form a hyperbolic curve. The lengths of red line and blue line are identical.

Figure 1 shows a simulated B-scan image of a point target located at $\left(x_{p},\;z_{p}\right)$ within the volume of a specimen. A short excitation laser pulse is irradiated at position $\left(x_{i},\;0\right)$ on the surface of the specimen and an acoustic wave is generated by the photoacoustic effect. The acoustic wave propagates into the volume with a spherical shape wavefront as shown in Figure 1a. In general, the excited acoustic wave has a short width due to the short duration of the exciting laser pulse. The echo ultrasound reflected or scattered by the point target located at $\left(x_{p},\;z_{p}\right)$ reaches the surface in another spherical wavefront. The echo signal measurements are assumed to be made at the same surface point for simplicity. In this case, when target point $\left(x_{p},\;z_{p}\right)$ is located far away from measuring point $\left(x_{i},\;0\right)$, the echo signal is measured after a significant time delay. Thus, the echo signal measured at various lateral positions appear with different delay times, and the plot of the time delay $t_{i}$ with respect to the lateral location $x_{i}$ of the measuring point provides a smooth curve as shown with the black solid line in Figure 1b. In the figure, the length of the red line (the distance to the target from measuring point $\left(x_{i},\;0\right)$) is the same as the length of the blue line (the equivalent distance in the vertical direction). The formation of equivalent distances at other lateral positions are depicted with black dotted lines in Figure 1b. It can be proved that the B-scan LUS image of a point-shape object appears as a smooth down-curved hyperbolic curve.

For a point target located at $\left(x_{p},\;z_{p}\right)$, the time delay of the short-enough LUS signal detected at a measuring point $\left(x_{i},\;0\right)$ is simply given as

(1) $$t_{i}=\frac{2}{v}\sqrt{{\left(x_{i}-x_{p}\right)}^{2}+{z_{p}}^{2}}$$

where v is the acoustic velocity and the factor 2 is due to the round trip. We can see that the time delay is minimum when $x_{i}$ is nearest to $x_{p}$ or just above the target. When the measurements are made continuously, at least from the mathematical point of view, Equation (1) can be written with continuous variables x and t as

(2) $$\frac{t^{2}}{a^{2}}-\frac{{\left(x-x_{p}\right)}^{2}}{b^{2}}=1$$

with two constants

(3) $$a=\frac{2}{v}z_{p}\;\mathrm{and}\;b=z_{p}$$

This is a typical equation for a hyperbolic curve. Therefore, adding the signals along the hyperbolic curve provides the intensity of the point target emanating the echo ultrasound. By superposing these target points, obtained with multiple hyperbolic curves in the B-scan image, the distribution of the target objects can be reconstructed.

### 2.2. Extraction of Acoustic Velocity

Equation (2) has the minimum time delay at $x=x_{p}$ of

(4) $$t_{\min}=a=\frac{2}{v}z_{p}$$

and the time delay becomes asymptotically proportional to x with

(5) $$t_{x>>x_{p}}=\frac{a}{b}\left(x-x_{p}\right)=\frac{2}{v}\left(x-x_{p}\right)$$

with these two characteristics, we can uniquely specify the hyperbolic curve if the acoustic velocity is known, and thus can determine the point target position $\left(x_{p},\;z_{p}\right)$.

**Figure 2:**
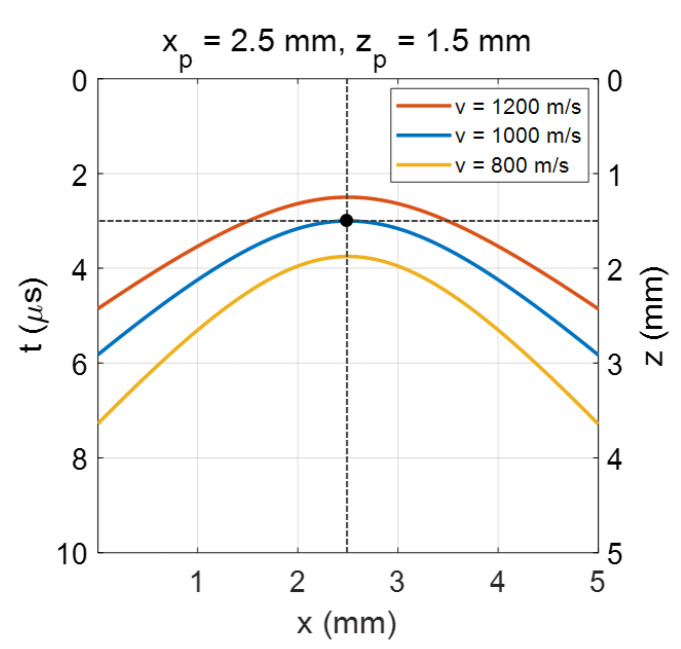
The hyperbolic curves simulated with the same target position but with different acoustic velocities. The minimum time delay and the slope of curve were changed with the velocity variation.

Numerical simulations have been performed to observe the general behaviors of the B-scan image of a point target with respect to the acoustic velocity of the medium surrounding the target. It was assumed that a point target was located at $\left(x_{p},\;z_{p}\right)$ = (2.5 mm, 1.5 mm) and the acoustic velocity was 1000 m/s. The B-scan image simulated with the correct velocity is plotted using the blue line in Figure 2. We can see the minimum time delay was obtained at $x_{p}$ = 2.5 mm, at which the depth was $z_{p}$ = 1.5 mm, the same as the assumed depth. As the lateral position deviated from $x_{p}$, the time delay increased. However, when the acoustic velocity was differently or wrongly assumed, the minimum time delay and the asymptotic increasing rate changed together. For example, with a 20% increased velocity (1200 m/s), the minimum time delay decreased from 3.0 μs to 2.5 μs, and the asymptotic slope flattened, as shown by the red curve in Figure 2. On the other hand, when a 20% decreased velocity was assumed (800 m/s), the minimum time delay was increased and the slope became stiffer, as shown by the yellow curve of the figure.

Therefore, we can say that the physically-same position of the target varies with the acoustic velocity of the medium. In other words, by adjusting the position of the point target only, the corresponding hyperbolic curve in its B-scan image cannot be constructed, meaning that the acoustic velocity must be specified or measured precisely before doing the reconstruction of a LUS signal. By performing a hyperbola curve fitting on the B-scan image, experimentally obtained with a simple target before the main measurements, we can obtain the acoustic velocity of the medium. In addition, any section of the target object with a point- or needle-like feature can be used to obtain the acoustic velocity. What is important to note is that the acoustic velocity of the object can be obtained without knowing the exact 3-dimensional location of the point target or the point-like feature.

### 2.3. Simulation of Target Object Reconstruction with T-SAFT

**Figure 3:**
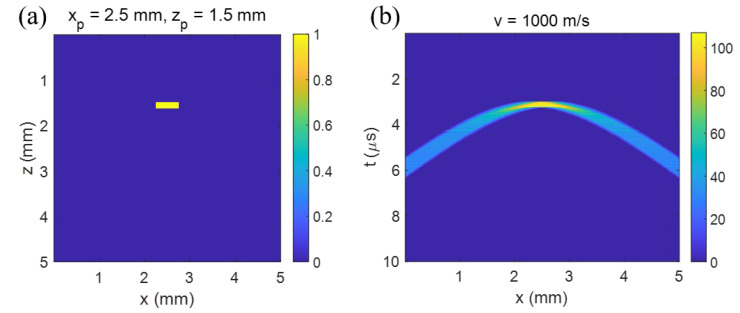
Simulation of LUS B-scan imaging. (**a**) The cross-sectional view of the target object. It is a line along *y*-axis but a uniform box in *x-z* plane. (**b**) The simulated B-scan image of the object in (**a**).

To simulate the reconstruction of the target object with T-SAFT, a B-scan image of a line shape target was assumed. The target object was assumed to be located at $\left(x_{p},\;z_{p}\right)$ = (2.5 mm, 1.5 mm) with a length of 500 μm along the *x*-axis and a width of 125 μm along the depth, as shown in Figure 3a. A uniform initial pressure distribution was assumed as shown with the color-bar in Figure 3a. The time delay $t_{i,p}$ was calculated from each target point located at $\left(x_{p},\;z_{p}\right)$, up to the surface measuring point, located at $\left(x_{i},\;0\right)$, using Equation (1). By superposing the data points $\left(x_{i},\;t_{i,p}\right)$ calculated for every point $\left(x_{p},\;z_{p}\right)$ composing the target object and scanning the measuring point laterally, the B-scan image of the object was obtained as seen in Figure 3b. When an acoustic velocity of 1000 m/s was used, we can see that each A-scan has a band along the time; this is due to the distribution of the points composing the target object.

Now, using the B-scan image, the image reconstruction of the original target object is simulated. The intensity $p_{0}$ at point $\left(x_{r},\;z_{r}\right)$ in the reconstructed image plane can be obtained by taking the overlap summation between the corresponding hyperbolic curve and the B-scan image. As was discussed above, the hyperbolic curve can be completely determined by the location of the target object and the acoustic velocity. Additionally, it can be obtained by adding all the intensities measured at every lateral scanning position $\left(x_{i},\;0\right)$ with the corresponding time delay $t_{i,r}$ given by Equation (1) as

(6) $$p_{0}(x_{r},z_{r})=\sum_{i=1}^{N}I_{i}(t_{i,r})=\sum_{i=1}^{N}I_{i}\left(\frac{2}{v}\sqrt{{\left(x_{i}-x_{r}\right)}^{2}+{z_{r}}^{2}}\right)$$

where N is the total number of A-scans used to obtain one B-scan image. 

**Figure 4:**
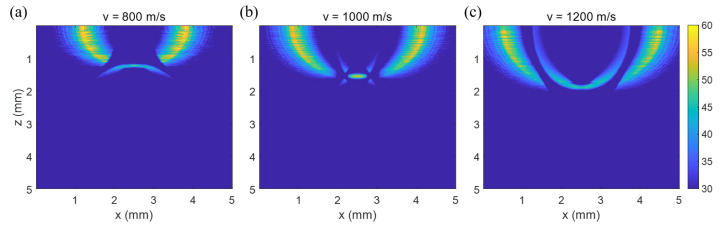
Image reconstruction of a LUS signal made with different acoustic velocities. The acoustic velocity was assumed (**a**) 800 m/s, (**b**) 1000 m/s, and (**c**) 1200 m/s. The image was well matched with the original when it was reconstructed with the velocity of 1000 m/s used to simulate the B-scan image.

To check the effect of acoustic velocity variation on the target object reconstruction, the target object from Figure 3b was reconstructed with three different acoustic velocities. As shown in Figure 4, the reconstructed image was heavily dependent on the acoustic velocity. When the acoustic velocity was equal to the one used to simulate Figure 3b, the reconstructed image was well matched with the original image (Figure 3a), especially the rod shape object in the middle of Figure 4b. However, when the velocity was slower than the ground truth value, the reconstructed target image was elongated and down-curved as shown in Figure 4a. Conversely, when the velocity was faster, it became up-curved as in Figure 4c. Accordingly, we can say that it is important to know the acoustic velocity of the medium for the reconstruction of LUS signal with a T-SAFT algorithm.

In addition, in Figure 4, we can see some artifacts in the shape of a bird wing that require further detailed investigation. One more noteworthy observation is that with this T-SAFT scheme, the intensity of a point in the reconstructed-image plane can be obtained using only a limited number of data points in the B-scan image plane. Only the data points used for the overlap summation with the corresponding hyperbolic curve are necessary. 

## 3. Experiments

### 3.1. Experimental Setup

**Figure 5:**
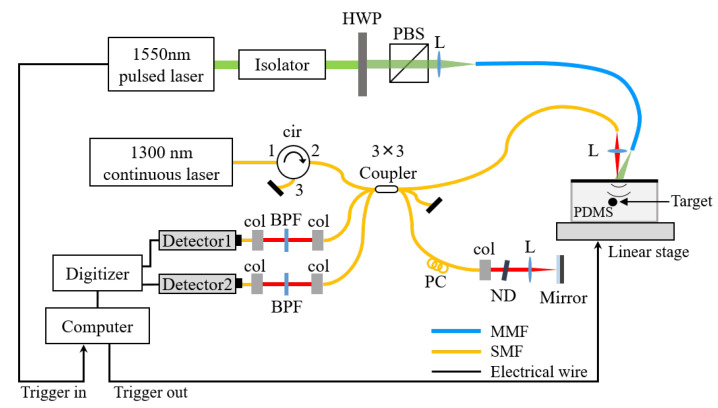
The LUS system implemented with a 3 × 3 optical interferometer. cir, circulator; PC, polarization controller; col, collimator; ND, neutral density filter; L, lens; BPF, band pass filter; HWP, half-wave plate; PBS, polarization beam splitter.

The LUS system was developed with a 3 × 3 optical interferometer, as shown with Figure 5. The intrinsic phase shift of a multiport interferometer was used to cope with the minute surface displacements induced by the acoustic echo signals. The detailed operation and principle of the 3 × 3 optical interferometer can be found in other studies [7,8].

**Figure 6:**
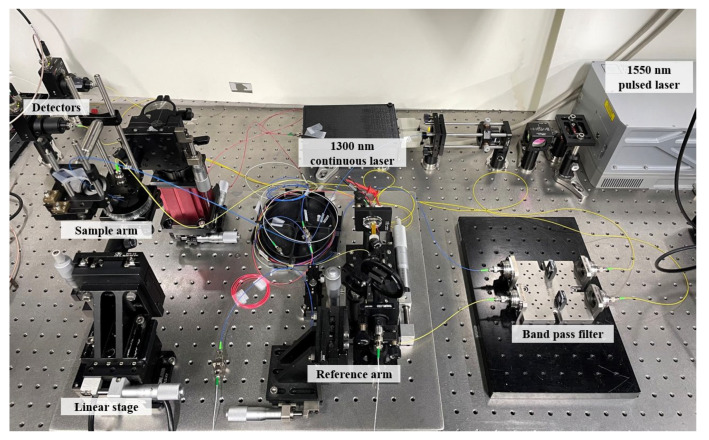
The photograph of developed all-optic LUS system. Both the excitation and the detection of acoustic waves are made with lasers.

A pulsed laser (Q-shift-B100-W1551, Quantum Light Instruments, Vilnius, Lithuania) with a wavelength of 1550 nm and a pulse width of 6.6 ns was used as the excitation beam. A half-wave plate and a polarization beam splitter were used to control the excitation intensity. A continuous laser (QDFBLD-1300-50, QPHOTONICS, Ann Arbor, MI, USA) with a wavelength of 1300 nm was used as the probe beam. The back-reflected light heading to the light source was blocked using an optical circulator. An optical band pass filter was used to prevent the probe beam from interfering with the strong excitation beam. The minute displacement that occurred in the sample arm was extracted from the signals detected at two return ports with the help of a computer. A photograph of the implemented LUS system is shown in Figure 6. The system was installed on an optical table with bulky components.

### 3.2. Sample Preparations

#### 3.2.1. Tissue Mimicking Phantom

**Figure 7:**
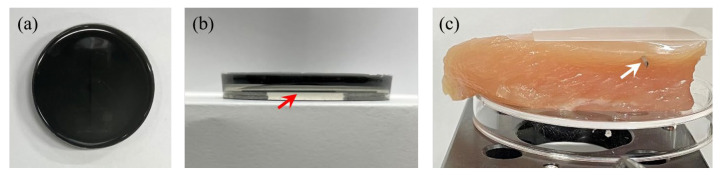
The specimens used for experiments. (**a**) Top view of a tissue-mimicking PDMS phantom and (**b**) its side view. A pencil lead with a 0.7 mm diameter, marked with the red arrow, was positioned at around 3.5 mm depth. (**c**) Chicken breast sample. A 1 mm diameter needle, marked with the white arrow, was positioned around 3.1 mm depth.

For the phantom medium mimicking a bio-tissue, a block of polydimethylsiloxane (PDMS) was prepared by mixing silicon with a curing agent in a ratio of 10:1. A pencil lead with a diameter of 0.7 mm was embedded into the PDMS block as the target object, as shown in Figure 7a. The pencil lead was positioned at a depth around 3.5 mm from the surface (as shown in Figure 7b marked with a red arrow), and then the PDMS resin was cured. To ensure the acoustic wave generation, a thin light absorption layer was placed on top of the pre-hardened PDMS block. The 1 mm thick absorption layer was produced by mixing carbon black powder, with a capacity of 1 mg/g, with the previously prepared PDMS mixture. The probe beam was focused on top of this absorption layer.

#### 3.2.2. Chicken Breast Sample

Chicken breast was used in order to acquire LUS signals from a bio-tissue specimen. A chicken breast was purchased at a grocery store and stored in a refrigerator until the experiment. Measurements were conducted at room temperature without any air conditioning. A 1 mm thick needle was inserted into the chicken breast at a depth around 3.1 mm, as shown in Figure 7c (marked with a white arrow). Since a laser with a wavelength of 1550 nm has high absorption in water, we did not place any additional absorption layer on the surface of chicken breast. However, to minimize the variation of light reflectance across the surface of the bio specimen, a cover glass with a thickness of 176 μm was attached to the surface with the help of ultrasound gel. Then, the surface displacement measurements were made at the outer surface of the cover glass.

## 4. Experimental Results

### 4.1. Tissue Mimicking PDMS Phantom

**Figure 8:**
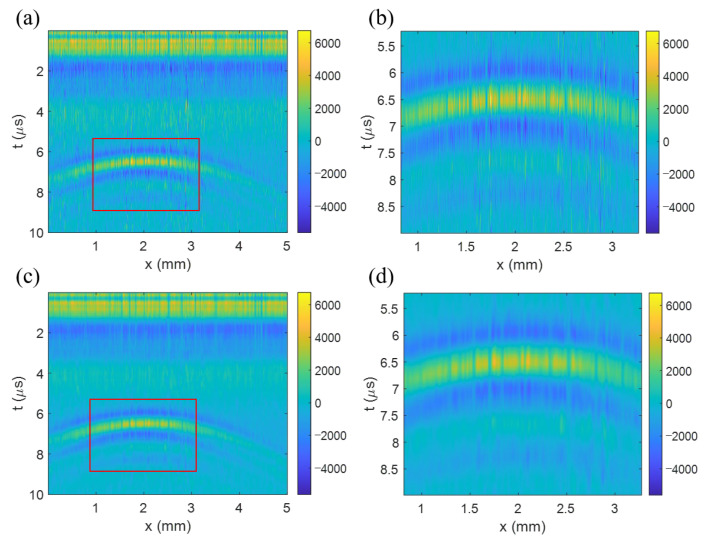
Experimentally acquired B-scan image of a PDMS block specimen. (**a**) Original image, (**b**) the enlarged image, (**c**) filtered image, and (**d**) the enlarged image. Gaussian filtering was used to remove the line shape noises in the vertical direction.

The surface displacements, induced by the echo ultrasound backscattered by the target object within the PDMS block, have been measured with the LUS system seen in Figure 6. The excitation beam was irradiated at the PDMS block with a 3 mm spot size and a probe beam with a 100 μm spot size was irradiated onto the top surface of the block. To obtain a B-scan image, the PDMS block was laterally shifted up to a span of 5 mm with a 5 μm step using a linear stage. As can be seen in Figure 8a, it was confirmed that the echo signal of the pencil lead was in a shape of hyperbola similar to the simulation result in Figure 3b. The curve was fitted with a hyperbolic curve to determine the position of the target point and the velocity of the acoustic wave traveling in the medium. To produce a more effective curve fitting, the highest pixel value in each A-scan was chosen around the area with the hyperbola-like signal feature. However, as shown in Figure 8b and the enlarged image of the red box in Figure 8a, there were severe line shape noises along the vertical direction. To remove this noise, a 2D Gaussian filter, using 20 × 20 pixels with a standard deviation of 2, was applied. Figure 8c, and its enlarged image Figure 8d, show that the vertical noises have been appreciably removed using the Gaussian filtering.

**Figure 9:**
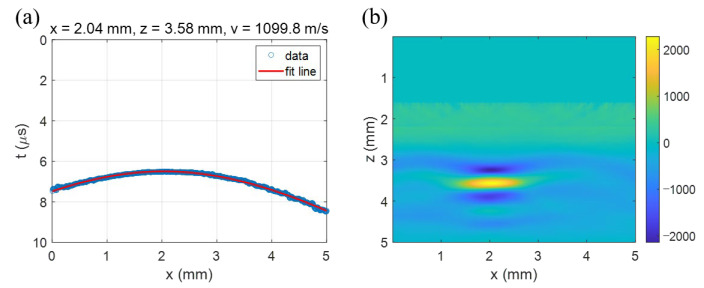
(**a**) The hyperbola curve fitting for extracting the acoustic velocity and (**b**) the T-SAFT image reconstruction made with the extracted velocity. The pencil lead initially placed at around 3.5 mm depth was reconstructed at 3.57 mm.

The hyperbola curve fitting produced using the filtered B-scan image (Figure 8c) gave the position of the target as $\left(x_{p},\;z_{p}\right)$ = (2.04 mm, 3.58 mm) and the acoustic velocity as 1099.8 m/s. Figure 9a shows that the fitted curve is well matched with the data points. By using the acoustic velocity extracted using the curve fitting, the T-SAFT image reconstruction was performed using the B-scan image in Figure 8. 

To reduce artifacts, the data within the time from 0 to 4 μs were nullified because the wanted surface displacement was irrelevant to the LUS signal in this domain. As a result, the target object was reconstructed as in Figure 9b. We can see that the image is well localized at a depth of around 3.57 mm. Though it is slightly elongated to the lateral direction, the image reconstructed with T-SAFT clearly identifies the target object (the pencil lead) placed at a depth of 3.5 mm, as presented in Section 3.2.1.

### 4.2. Chicken Breast Sample

**Figure 10:**
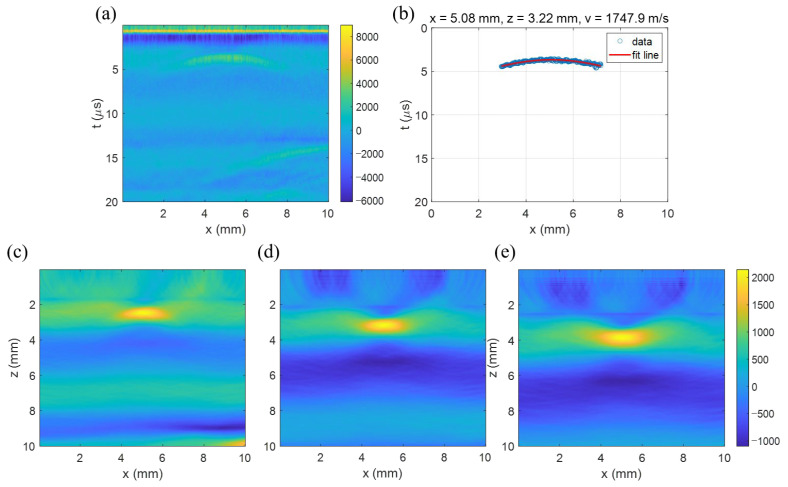
T-SAFT image reconstruction of the chicken breast sample. (**a**) The experimentally acquired B-scan image. (**b**) The hyperbola curve fitting result, which gave the acoustic velocity as 1747.9 m/s. The image reconstruction made with a (**c**) lower velocity of 1398.3 m/s, (**d**) proper velocity of 1747.9 m/s, and (**e**) higher one of 2097.4 m/s. The images are distorted with wrong velocities.

As a bio-tissue specimen, the chicken breast, embedded with a needle, was B-scanned with a lateral span of 10 mm and a 10 μm step. Figure 10a shows that the B-scan image has a hyperbola-like curve, though it is slightly faint. The curve was fitted with a hyperbolic curve in the domain from 2.4 mm to 7.0 mm, as shown in Figure 10b, which gave the target position as $\left(x_{p},\;z_{p}\right)$ = (5.08 mm, 3.22 mm) and the acoustic velocity as 1747.9 m/s. To reduce artifacts during the reconstruction process, the data within the time from 0 to 1.2 μs of the B-scan image were nullified once again. Figure 10d shows the image reconstructed using the extracted acoustic velocity of 1747.9 m/s. We can see the well-localized image of the needle at a depth of around 3.1 mm. To investigate the effect of acoustic velocity, the reconstruction was made using other velocities. Figure 10c was produced using a 20% lower velocity (1398.3 m/s) and Figure 10e using a 20% higher velocity (2097.4 m/s). Using the lower velocity, the down curved image was observed as with the simulation result in Figure 4a. Meanwhile, using the higher velocity produced the up curve as with the simulation in Figure 4c. Certainly, the proper velocity provided the mostly-localized reconstruction image of the needle as expected. In addition, the measurements suggest that the depth of the reconstructed image also depends on the velocity. Using the lower velocity, the depth was 2.44 mm, but with the higher velocity, it was about 3.87 mm, far from the actual depth of 3.17 mm.

## 5. Discussions

The velocity of acoustic wave propagation in a medium varies primarily with the composing material but can it also be affected by environmental conditions [28,29,30]. In the case of the PDMS phantom, the velocity depends on the mixing ratio and the baking temperature and duration [31,32]. For bio-samples, the velocity varies from sample to sample, and the medium is not uniform in space nor stable in time. It also varies with temperature and humidity at the time of measurement. Thermal coagulation of a bio-tissue, likely due to the excitation laser beam, changes the acoustic velocity [29,33]. Accordingly, it is important to determine the acoustic velocity in the actual environment or in situ.

For measuring acoustic velocity, the area having a hyperbola-like feature is selected in the B-scan image and then a curve is extracted from the area. Then, the curve fitting is applied to the extracted curve. In our case, a mask was applied to the area with the hyperbola feature in the B-scan image and the pixel with the highest value in each A-scan was selected. Finally, the curve fitting was applied to the curve composed of the selected data points. The hyperbola curve fitting provides the location of the point target and the acoustic velocity of the medium embedding the target simultaneously. Therefore, prior knowledge of the exact location of the point target is not necessary to extract the acoustic velocity. In experiments, the point target can be intentionally embedded in the specimen, as with the pencil lead and needle in this study, but a point- or needle-like feature on the target object can be used to obtain the acoustic velocity.

When the approximate location and shape of the target object are known, overlap additions can be produced in a limited area, reducing the processing time significantly. However, for a general case in which the distribution of objects within a specimen is not available before measurement, the overlap addition method is not preferable. In this case, the intensity at a point in the reconstructed-image plane can be made by adding the contributions from all the data points in the B-scan image. For an example, the signal at a point $\left(x_{i},\;t_{i}\right)$ in the B-scan image appears as a circle in the reconstructed plane, centered at $\left(x_{i},\;0\right)$ with a radius of ri=12vti. By adding the circles, the reconstruction across the whole plane can be conducted. If the point has a greater number of overlapping circles, it will have a stronger intensity. Even though the process requires all the data points in the B-scan image plane for reconstructing just one point in the reconstructed-image plane, it is straightforward and simple. If we can identify some convincing features in a B-scan image, we can reconstruct the full image with a limited dataset.

For the bio specimen, such as the chicken breast, the acoustic wave generating layer was not intentionally added because the bio-tissue had a rather large rate of light absorption at 1550 nm. However, by using a cover plate having a high light absorption at one side of the plate, we might increase the efficiency of acoustic wave generation. A coating material with high light absorption at near infrared was developed using cesium tungsten bronze [34]. We can also think about using carbon nanotubes and/or graphene materials for our further study.

For a real field application, extensive system engineering jobs are expected, including packaging and optimizing. We are planning to implement an endoscopic system for biomedical applications soon.

## 6. Conclusions

LUS signals were acquired in a non-contact manner from a tissue-mimicking PDMS phantom and a chicken breast using a developed all-optic LUS system. The exciting acoustic wave was generated at the surface of a specimen with a pulsed laser. The echo signal returned by the target object in the specimen was measured at the surface using a different laser. The small displacement induced by the echo ultrasound could be measured with the interferometer implemented with a 3 × 3 fiber coupler. It was confirmed that a point-like target object within a specimen was observed at a fair extent of lateral scanning; thus, it appeared as a smooth curve in its B-scan image. The curve was fitted well with a hyperbolic curve. From this fitted curve, both the position of the object and the velocity of the acoustic wave could be extracted simultaneously.

To reconstruct the initial target object from the B-scan image, T-SAFT was used, in which the echo signals returned by a single target point, but measured at several lateral positions, have been coherently added together. It was confirmed that the intensity at a particular point in the reconstructed-image plane could be obtained by taking the overlap integral or addition between the B-scan image with the hyperbolic curve determined by the position of the point. However, without knowing the proper acoustic velocity, the T-SAFT reconstruction could not be made successfully.

After the hyperbola curve fitting, the acoustic velocities of the PDMS and chicken breast sample were obtained as 1099.8 m/s and 1747.9 m/s in our experimental condition, respectively. The T-SAFT produced using these velocities successfully reconstructed the image of the pencil lead and the needle embedded in each specimen. In addition, it was observed that the incorrectly estimated faster velocity falsely identified the target at a deeper position in the reconstructed-image plane; likewise, the slower velocity determined the target to be at a shallower position. In conclusion, we can say that the proposed curve fitting method not only provides the in situ acoustic velocity of the specimen under harsh or unwanted environmental conditions but also allows us to obtain a more precise tomographic imaging of a 3-dimensional target object.

## Data Availability

The data presented in this study are available on request from the corresponding author. The data are not publicly available due to privacy or ethical restrictions.
